# MicroRNA-137 Inhibited Hypoxia-Induced Proliferation of Pulmonary Artery Smooth Muscle Cells by Targeting Calpain-2

**DOI:** 10.1155/2021/2202888

**Published:** 2021-09-01

**Authors:** Xiao-Yue Ge, Tian-Tian Zhu, Mao-Zhong Yao, Hong Liu, Qian Wu, Jie Qiao, Wei-Fang Zhang, Chang-Ping Hu

**Affiliations:** ^1^Department of Pharmacology, Xiangya School of Pharmaceutical Sciences, Central South University, Changsha, 410078 Hunan, China; ^2^Teaching and Research Office of Clinical Pharmacology, College of Pharmacy, Xinxiang Medical University, Xinxiang, 453003 Henan, China; ^3^Department of Pharmacy, The Second Affiliated Hospital of Nanchang University, Nanchang, 330006 Jiangxi, China; ^4^Human Provincial Key Laboratory of Cardiovascular Research, Central South University, Changsha, 410078 Hunan, China

## Abstract

The proliferation of pulmonary artery smooth muscle cells (PASMCs) is an important cause of pulmonary vascular remodeling in pulmonary hypertension (PH). It has been reported that miR-137 inhibits the proliferation of tumor cells. However, whether miR-137 is involved in PH remains unclear. In this study, male Sprague-Dawley rats were subjected to 10% O_2_ for 3 weeks to establish PH, and rat primary PASMCs were treated with hypoxia (3% O_2_) for 48 h to induce cell proliferation. The effect of miR-137 on PASMC proliferation and calpain-2 expression was assessed by transfecting miR-137 mimic and inhibitor. The effect of calpain-2 on PASMC proliferation was assessed by transfecting calpain-2 siRNA. The present study found for the first time that miR-137 was downregulated in pulmonary arteries of hypoxic PH rats and in hypoxia-treated PASMCs. miR-137 mimic inhibited hypoxia-induced PASMC proliferation and upregulation of calpain-2 expression in PASMCs. Furthermore, miR-137 inhibitor induced the proliferation of PASMCs under normoxia, and knockdown of calpain-2 mRNA by siRNA significantly inhibited hypoxia-induced proliferation of PASMCs. Our study demonstrated that hypoxia-induced downregulation of miR-137 expression promoted the proliferation of PASMCs by targeting calpain-2, thereby potentially resulting in pulmonary vascular remodeling in hypoxic PH.

## 1. Introduction

Pulmonary hypertension (PH) is a rare vascular disorder, now defined clinically as a mean pulmonary artery pressure (mPAP) over 25 mmHg at rest or over 30 mmHg during activity. Pulmonary vascular remodeling plays an important role in PH pathology, which is mainly characterized by endothelial cell injury, smooth muscle cell proliferation, fibroblast muscularization, extracellular matrix increase, in situ thrombosis, varying degree inflammation, and plexiform arterial changes [1, 2]. In these pathological changes, the proliferation of pulmonary arterial smooth muscle cells (PASMCs) is the most important cause of pulmonary vascular remodeling in PH. Therefore, inhibition of PASMC proliferation is expected to be a crucial pathway for PH treatment.

Calpain is a Ca^2+^-dependent cysteine protease that has been found to contain at least 15 subtypes, calpain-1 (*μ*-calpain) and calpain-2 (m-calpain), which are the two best-characterized members of the calpain family and are ubiquitously expressed in mammals [3]. Calpain-1 and calpain-2 constitute a distinct larger catalytic subunit, and calpain-4 as a common smaller subunit is responsible for maintaining calpain activity [4]. Recent studies have linked calpain with a variety of diseases, such as Alzheimer's and Parkinson's diseases, cancer, diabetes, atherosclerosis, and PH [5]. In hypoxia and monocrotaline-induced PH of mice and rats, the expression of calpain-1/2/4 in the lung tissues and pulmonary arteries was significantly increased [6–8]. Research focusing on the role of calpain-2 in hypoxia-induced PH becomes a meaningful work.

It has been reported that a variety of miRNAs participate in the pathogenesis of PH. For example, miR-223 [9] and miR-let-7g [10] have been found to regulate the proliferation of PASMCs participating in pulmonary vascular remodeling of PH. To fully reveal the role of miRNAs in hypoxic PH, we did the pilot microarray assay in pulmonary arteries of hypoxic PH rats and found that the expression of miR-137 was significantly downregulated. It has been reported that miR-137 inhibits the proliferation and migration of a variety of tumor cells [11, 12]. Over 1000 genes have been predicted to be targets of miR-137 by using a bioinformatic approach, and highlighted target genes are involved in a large number of pathways including neural development, cell cycle, differentiation, and proliferation [13]. However, whether miR-137 is involved in PH remains unclear. Bioinformatic analysis suggests that the 3′-UTR of calpain-2 contains a potential binding element for miR-137 with a 7-nt match to the miR-137 seed region, and miR-137 has been found to directly target calpain-2 in motoneurons [14]. We therefore hypothesize that miR-137 contributes to hypoxic PH by targeting calpain-2 and designed this study to explore the regulatory role of miR-137 in hypoxia-induced PASMC proliferation and pulmonary arterial remodeling in rat hypoxic PH, and the regulating effect of miR-137 on calpain-2 expression was also certificated.

## 2. Materials and Methods

### 2.1. Animal Experiments

About 180-220 g, male Sprague-Dawley (SD) rats were purchased from the Laboratory Animal Center of Xiangya School of Medicine, Central South University, Changsha, China (SCXK (XIANG) 2019-0014). All protocols of animal experiments (No. CSU2017009) were approved by the Central South University Veterinary Medicine Animal Care and Use Committee. Regarding the methodology, we followed the PH preclinical guidelines as previously described [15].

SD rats were randomly divided into hypoxia group and control group. Rats were exposed to continuity hypoxia (10% O_2_) for up to 21 days in the hypoxia group while maintained in a normal oxygen condition (21% O_2_) in the control group. At the 21 days after subjected to hypoxia, the rats were weighed and anesthetized by intraperitoneal injection of 2% sodium pentobarbital (60 mg/kg). A Vevo 2100 (VisualSonics, Canada) ultrasound system equipped with 21 MHz probe was used for echocardiographic assessment of pulmonary arterial acceleration/ejection time ratio (PAAT/PAET). Right-sided heart catheterization was conducted to detect right ventricular systolic pressure (RVSP) and mPAP. The right ventricle (RV) was separated from left ventricle and septum (LV+S) and weighed. The ratio of RV to (LV+S) was calculated to assess the extent of right ventricle hypertrophy. The pulmonary arterial samples were collected for mRNA and protein expression analysis. The right lower lung was fixed in 4% paraformaldehyde for hematoxylin-eosin (HE) staining and in situ hybridization analysis of miR-137.

### 2.2. HE Staining

For HE staining, the fixed lungs were embedded in paraffin and then cut into approximately 5 *μ*m thick sections by microtome. HE staining of right lung was conducted in accordance to the same method used in our previous study [6].

### 2.3. In Situ Hybridization

In situ hybridization kit (Boster, Wuhan, China) was used to detect the expression of miR-137 in lung tissues according to the manufacturer's instructions. In brief, 5 *μ*m sections were used for sodium citrate antigen retrieval and then incubated with blocking buffer overnight with miR-137 detection probe which was labeled with 3′ and 5′ digoxigenin. After washed with phosphate-buffered saline (PBS) and SSC buffer, immunodetection was performed with a biotinylated anti-DIG antibody at 37°C for 60 min and the avidin-biotin-peroxidase complex (ABC kit, Vector Laboratories, Burlingame, CA) at 37°C for 20 min. After washed with PBS, the slides were detected by 3,3-diamino benzidine (DAB) staining.

### 2.4. Preparation of Primary Rat PASMCs

As our previous study described, primary rat PASMCs were extracted from the pulmonary arteries using tissue block anchorage method [10]. Dulbecco's modified Eagle's medium (DMEM) supplemented with 20% (v/v) fetal bovine serum was used to culture primary rat PASMCs at 37°C in a humidified atmosphere of 5% CO_2_. Smooth muscle *α*-actin (*α*-SMA) immunohistochemistry and immunofluorescence using anti-rat *α*-SMA antibody (1 : 50, ab7817, Abcam) were used to identify PASMCs. The three to five passages of PASMCs were used for all experiments.

### 2.5. Cell Transfection

PASMCs reached 60% to 70% of confluence were starved with low serum sputum (2% FBS) for 24 h. To validate the effects of miR-137 and calapin-2 on hypoxia-induced PASMC proliferation and gene expression, the mimic and inhibitor of miR-137 and calpain-2 siRNA (Ribobio Co. Ltd., Guangzhou, China) were transiently transfected by ribo FECT™ CP transfection kit (Ribobio Co. Ltd., Guangzhou, China) according to the manufacturer's instructions. Then, the cells were maintained in hypoxia (3% O_2_) or normoxia chamber for up to 48 h according to grouping. Quantitative real-time polymerase chain reaction was used to detect the transfection efficiency of miR-137 mimic. Real-time PCR and Western blot were used to test the expression of calpain-2 mRNA and protein to detect the transfection efficiency of calpain-2 siRNA. The target sequences of calpain-2 siRNAs were CCAATTTGTTCAAGATCAT.

### 2.6. Assay of Cell Proliferation

For the MTS assay as described previously [10], PASMCs were seeded in 96-well culture plates (6 × 10^3^ cells/well) and then starved with low serum sputum (2% FBS) for 24 h. After treatment, the cells were washed with PBS. According to the manufacturer's instructions, each well was added 10 *μ*L of MTS solution and incubated at 37°C for 2.5 h after the treatment. Colorimetric analysis was determined by an ELISA plate reader (DTX880; Beckman, Miami, FL) at 490 nm.

For EDU proliferation assay, 5 × 10^3^ cells/well were seeded into 96-well culture plates. According to the manufacturer's instructions, each well was added 50 *μ*mol/L of 5-ethynyl-2′-deoxyuridine (EDU, Ribobio, China) and incubated at 37°C for 4 h. The cells were fixed by using 4% formaldehyde for 15 min and then treated with 50 *μ*L 2 mg/mL glycine for 5 min at 25°C. Then, the cells were treated with 100 *μ*L 0.5% TritonX-100. After washing with PBS for 3 times, 100 *μ*L of 1×Apollo® reaction cocktail was added in each well and reacted for 30 min. Then, the cells were stained with 100 *μ*L of Hoechst 33342 (5 *μ*g/mL) for 30 min and visualized under a fluorescent microscope.

### 2.7. RNA Isolation and Real-Time PCR Analysis

The mRNA levels of miR-137 and calpain-2 were quantified by real-time PCR. In brief, total RNA of pulmonary arteries and PASMCs was extracted by TRIzol reagent (Invitrogen, Carlsbad, CA) and the concentration and quality of RNA were confirmed by spectrophotometric method. Prime Script reverse transcription reagent Kit (DRR037S; TaKaRa) was used for RNA reverse transcription reaction. ABI Prism 7300 real-time PCR system (Applied Biosystems) with SYBR Premix Ex Taq (DRR041A; TaKaRa) was used for quantitative analysis of mRNA expression. Primers for calpain-2: (F) CCAGAAGTTGGTGAAAGGACA and (R) CTGCCGTTCTGTTAGATTTGC and *β*-actin: (F) TGTCACCAACTGGGACGATA and (R) ACCCTCATAGATGGGCACAG. For the detection of miR-137, Bulge-Loop miRNA Primers (Ribobio) were replaced oligo and random primers during reverse transcription reaction. Data analysis was performed by comparative *C*t method using the ABI software. *β*-Actin and U6 were used to normalize the expression level of mRNAs and miRNAs, respectively.

### 2.8. Reverse Transcriptase Polymerase Chain Reaction (RT-PCR)

Preparation of cDNA was carried out from 2 *μ*g of total RNA using the TranScript One-Step gDNA Removal and cDNA Synthesis SupperMix for RT-PCR (TransGen Biotech, China) according to the manufacturer's instructions. Semiquantitative RT-PCR cDNA was amplified in a 25 *μ*L reaction volume containing 2.5 mM dNTPs, 10 *μ*M specificprimers, 10×EasyTag buffer, and 1 U of EasyTag DNA Polymerase (TransGen Biotech, China). After initial denaturation at 94°C for 5 min, PCR was carried out for 35 cycles with denaturation for 30 s at 94°C, annealing for 30 s at 56°C for PCNA and beta-actin, and extension for 1 min at 72°C followed by afinalextension of 10 min at 72°C. Primers for PCNA: (F) TACAAGCAACTTCCCATTCCA and (R) TCAGCAAACACAACTCCTCCT and *β*-actin: (F) CCCATCTATGAGGGTTACGC and (R) TTTAATGTCACGCACGATTTC. The PCR products were visualized by electrophoresis with an ethidium bromide-stained 1.5% agarosegel. The densitometric analysis was conducted with UVP Bioimaging System (BioDoc, USA).

### 2.9. Western Blot Analysis

Proteins were extracted from cultured PASMCs and pulmonary arteries with RIPA buffer (contain 1% PMSF) for 30 min on ice and quantified by BCA kit (P0010, Beyotime, China). About 20~60 *μ*g protein of each sample was separated by 10% SDS-polyacrylamide gels and transferred onto PVDF membranes. Membranes were blocked with 5% skim milk for 1 h and then incubated with primary antibodies for calpain-2 (ab39165, Abcam, 1 : 1000), PCNA (A0264, ABclonal, 1 : 1000), and *β*-actin (AF0003, Beyotime, 1 : 1000) and subsequently incubated with horseradish peroxidase- (HRP-) coupled goat anti-rabbit (A0208, Beyotime, 1 : 1000) and HRP-coupled goat anti-mouse (A0216, Beyotime, 1 : 1000). The chemiluminescence signals were visualized with the LuminataTM Crescendo substrate (WBLUR0100, Millipore). The densitometric analysis was conducted with ChemiDoc XRS+ system (Bio-Rad Co. Ltd., USA).

### 2.10. Luciferase Assay

The 3′-UTR of calpain-2 mRNA with putative/mutant miR-137 binding site was cloned into the firefly luciferase reporter construct pmiR-RB-ReportTM Vector (Ribobio, Guangzhou, China). Firefly luciferase (Luc) acts as a control, and renilla luciferase (Rluc) acts as a reporter. For the reporter assay, PASMCs grown in 96-well plates were cotransfected with calpain-2-3′-UTR-Luc (2 *μ*g) and miR-137 mimic (50 nM) by ribo FECT™ CP transfection kit. Dual-Luciferase® Reporter Assay System (E1910, Promega) was used to detect the renilla and firefly luciferase activities after incubation for 48 h.

### 2.11. Statistics

Data were shown as mean ± S.E.M.(standard errors). Statistical analysis was performed by the permutation test when the sample size is only 3 and by Student's *t*-test for two groups or by one-way ANOVA followed by Student-Newman-Keuls test for multiple groups when the sample size is greater than 3. A value of *p* less than 0.05 was considered to be statistically significant. All statistical analyses were performed by the SPSS18.0 software, and GraphPad Prism 7 was used for drawing figures.

## 3. Results

### 3.1. miR-137 Was Downregulated in Remodeled Pulmonary Arteries and Hypoxia-Treated PASMCs in Hypoxic PH

**Figure 1:**
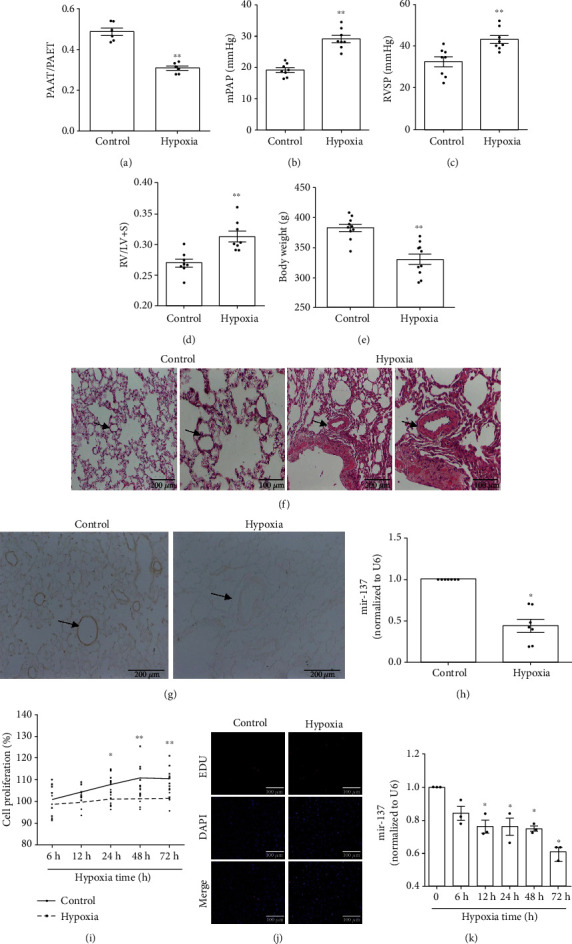
miR-137 was downregulated in remodeled pulmonary arteries and hypoxia-treated PASMCs. (a) PAAT/PAET (*n* = 6). (b) mPAP (*n* = 8). (c) RVSP (*n* = 8). (d) RV/LV+IS (*n* = 8). (e) Body weight of rats (*n* = 10). (f) HE staining. (g, h) The expression of miR-137 in rat pulmonary arteries was detected by in situ hybridization and real-time PCR (*n* = 7). (i) The proliferation of PASMCs was detected by MTS assay (*n* = 5). (j) The proliferation of PASMCs was detected by EDU staining. (k) The expression of miR-137 in the PASMCs after hypoxia stimulation for 0, 6, 12, 24, 48, and 72 h was measured by real-time PCR (*n* = 3). PAAT/PAET: pulmonary arterial acceleration/ejection time ratio; mPAP: mean pulmonary arterial pressure; RVSP: right ventricular systolic pressure; RV: right ventricle; LV: left ventricle; IS: the interventricular septum. The data are presented as means ± S.E.M.; ^∗^*p* < 0.05 and ^∗∗^*p* < 0.01 vs. 0 h, control.

To induce hypoxic PH, the rats were exposed to hypoxia (10% O_2_) for 21 days. As keeping with our previous study [10], PAAT/PAET (Figure 1(a)) was markedly decreased in the hypoxia group; meanwhile, mPAP (Figure 1(b)), RVSP (Figure 1(c)), and the right heart remodeling index RV/(LV+S) (Figure 1(d)) were significantly increased in the hypoxia group. The body weight of hypoxic PH rats was decreased compared with the control group (Figure 1(e)). HE staining demonstrated that hypoxia induced obvious thickening of the pulmonary vascular wall and the stenosis of the lumen (Figure 1(f)).

Accordance to our pilot study based on the microarray assay (mentioned in Introduction), the expression of miR-137 was measured in pulmonary arteries and PASMCs. As shown in Figures 1(g) and 1(h), hypoxia significantly downregulated the expression of miR-137 in pulmonary arteries of hypoxic PH rats. As expected, PASMCs exposed to 3% O_2_ for different times (6 h, 12 h, 24 h, 48 h, and 72 h) showed significant proliferation in a time-dependent manner (Figures 1(i) and 1(j)). With the proliferation of hypoxia-induced PASMCs, hypoxia also significantly downregulated the expression of miR-137 in PASMCs (Figure 1(k)).

### 3.2. miR-137 Inhibited Hypoxia-Induced Proliferation of PASMCs

**Figure 2:**
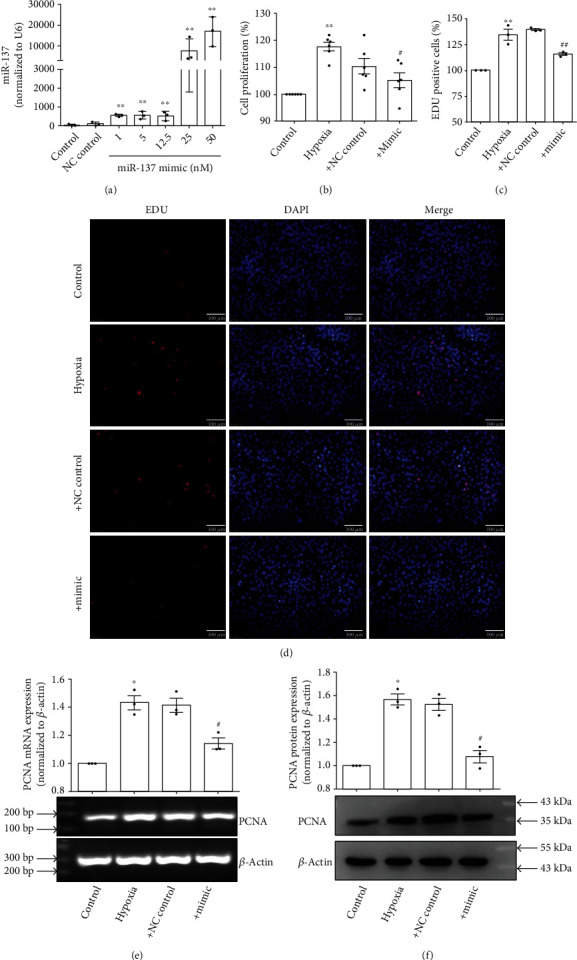
miR-137 inhibited hypoxia-induced proliferation of PASMCs. (a) PASMCs were transfected with miR-137 mimic, and the expression of miR-137 was detected by real-time PCR (*n* = 3). (b) PASMCs were transfected with miR-137 mimic (25 nM), and the proliferation of PASMCs was detected by MTS assay (*n* = 6). (c) Statistic diagram of EDU staining (*n* = 3). (d) PASMCs were transfected with miR-137 mimic (25 nM), and the proliferation of PASMCs was detected by EDU staining. (e) PASMCs were transfected with miR-137 mimic (25 nM), and RT-PCR was used to detect the mRNA expression of PCNA, a marker of cell proliferation (*n* = 3). (f) PASMCs were transfected with miR-137 mimic (25 nM), and Western blot detected the protein expression of PCNA (*n* = 3). The data are presented as means ± S.E.M.; ^∗^*p* < 0.05 and ^∗∗^*p* < 0.01 vs. control and ^#^*p* < 0.05 and ^##^*p* < 0.01 vs. hypoxia.

As mentioned above, miR-137 regulates the proliferation of a variety of tumor cells [11, 12]. We therefore explored the regulatory effect of miR-137 on hypoxia-induced proliferation of PASMCs by transfecting the mimic of miR-137. The results demonstrated that the transfection of miR-137 mimic significantly increased the expression of miR-137 (Figure 2(a)) and remarkably relieved hypoxia-induced the proliferation of PASMCs (Figures 2(b)–2(f)).

### 3.3. miR-137 Inhibitor Induced the Proliferation of PASMCs

**Figure 3:**
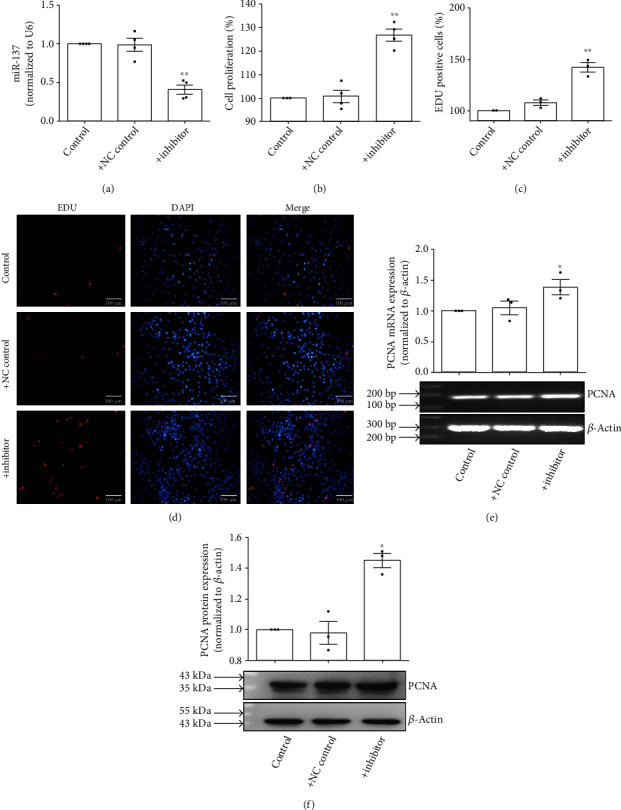
miR-137 inhibitor induced the proliferation of PASMCs. (a) PASMCs were transfected with miR-137 inhibitor (100 nM), and the expression of miR-137 was detected by real-time PCR (*n* = 4). (b) PASMCs were transfected with miR-137 inhibitor (100 nM), and the proliferation of PASMCs was detected by MTS assay (*n* = 4). (c) Statistic diagram of EDU staining (*n* = 3). (d) PASMCs were transfected with miR-137 inhibitor (100 nM), and the proliferation of PASMCs was detected by EDU staining. (e) PASMCs were transfected with miR-137 inhibitor (100 nM), and RT-PCR was used to detect the mRNA expression of PCNA (*n* = 3). (f) PASMCs were transfected with miR-137 inhibitor (100 nM), and Western blot detected the protein expression of PCNA (*n* = 3). The data are presented as means ± S.E.M.; ^∗^*p* < 0.05 and ^∗∗^*p* < 0.01 vs. control.

To further confirm the role of miR-137 in the proliferation of PASMCs, we transfected the inhibitor of miR-137 (100 nM) to PASMCs under normoxia. As Figure 3 shown, miR-137 inhibitor decreased the expression of miR-137 (Figure 3(a)) and induced the proliferation of PASMCs (Figures 3(b)–3(f)).

### 3.4. Hypoxia Induced the Expression of Calpain-2

**Figure 4:**
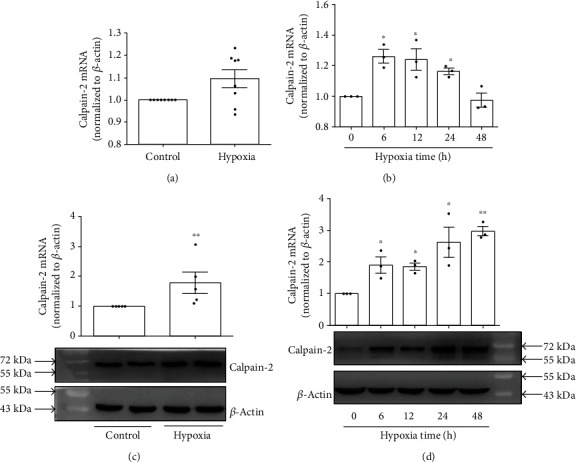
Calpain-2 was upregulated in remodeled pulmonary arteries and hypoxia-treated PASMCs. (a) The mRNA expression of calpain-2 in the pulmonary arteries of rats (*n* = 8). (b) The protein expression of calpain-2 in the pulmonary arteries of rats (*n* = 8). (c) The mRNA expression of calpain-2 in PASMCs (*n* = 3). (d) The protein expression of calpain-2 in PASMCs (*n* = 3). The data are presented as means ± S.E.M.; ^∗^*p* < 0.05 and ^∗∗^*p* < 0.01 vs. 0 h, control.

It has well been documented that calpain-2 is mediated in promoting the proliferation of PASMCs, thereby resulting to pulmonary arterial remodeling in hypoxic PH [6–8]. In our setting, we therefore measured the expression of calpain-2 and found that exposure of rats to continuity hypoxia (10% O_2_) for 21 days significantly upregulated the protein expression of calpain-2 in pulmonary arteries (Figure 4(b)) but not the expression of calpain-2 mRNA meanwhile (Figure 4(a)). Accordantly, treatment of PASMCs with 3% O_2_ for 6 h, 12 h, 24 h, and 48 h also upregulated the mRNA and protein expression of calpain-2 in a time-dependent manner (Figures 4(c) and 4(d)).

### 3.5. miR-137 Inhibited Hypoxia-Induced Upregulation of Calpain-2 Expression

**Figure 5:**
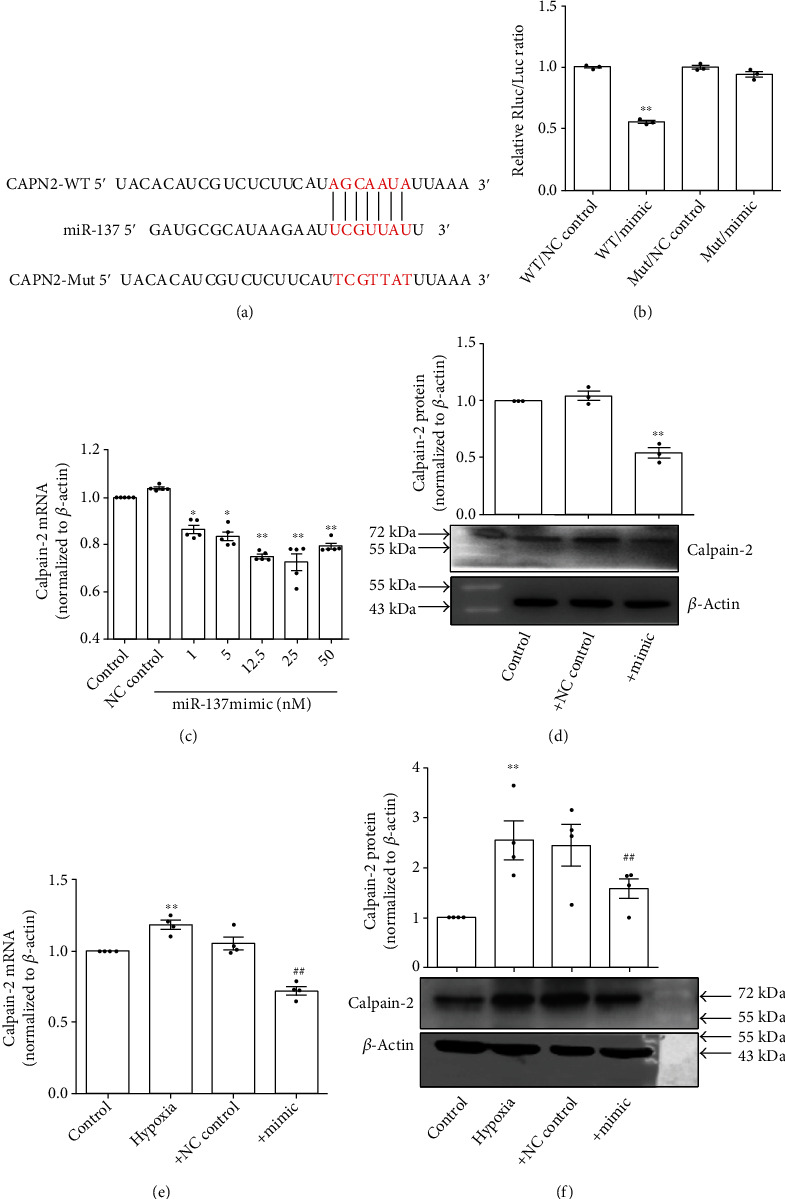
miR-137 inhibited hypoxia-induced upregulation of calpain-2 expression. (a) The putative binding site of miR-137 in 3′-UTR of calapin-2 mRNA. (b) Luciferase analysis for examining whether miR-137 targets 3′-UTR of calpain-2 mRNA (*n* = 3). (c) The mRNA expression of calpain-2 in PASMCs after transfecting miR-137 mimic under normoxic condition (*n* = 5). (d) The protein expression of calpain-2 in PASMCs after transfecting miR-137 mimic (25 nM) under normoxic condition (*n* = 3). (e) The mRNA expression of calpain-2 in PASMCs after transfecting miR-137 mimic (25 nM) under hypoxic condition (*n* = 4). (f) The protein expression of calpain-2 in PASMCs after transfecting miR-137 mimic (25 nM) under hypoxic condition (*n* = 4). WT: wild type; Mut: mutant; NC: negative control. The data are presented as means ± S.E.M.; ^∗^*p* < 0.05 and ^∗∗^*p* < 0.01 vs. control or WT+NC control and ^##^*p* < 0.01 vs. hypoxia.

It has been documented that miR-137 inhibits the mRNA of calpain-2 by directly targeting at 3′-UTR of calpain-2 [14, 16]. To explore whether miR-137 targets 3′-UTR of calpain-2 mRNA in PASMCs, we mutated the putative binding site (Figure 5(a)). As shown in Figure 5(b), miR-137 mimic significantly downregulated the fluorescence values of wild-type vectors, whereas luciferase activity was unchanged using 3′-UTR binding site-mutated construct. These results indicated that miR-137 repressed the translation of calpain-2 mRNA by binding to its 3′-UTR. We then observed the effect of the transfection of miR-137 mimic on the expression of calpain-2 in PASMCs and found that miR-137 mimic downregulated the expression of calpain-2 mRNA and protein expression under normoxic condition (Figures 5(c) and 5(d)). It is of note that miR-137 mimic (25 nM) reversed the upregulated expression of calpain-2 (both mRNA and protein) induced by hypoxia (Figures 5(e) and 5(f)).

### 3.6. Knockdown of Calpain-2 Inhibited Hypoxia-Induced PASMC Proliferation

**Figure 6:**
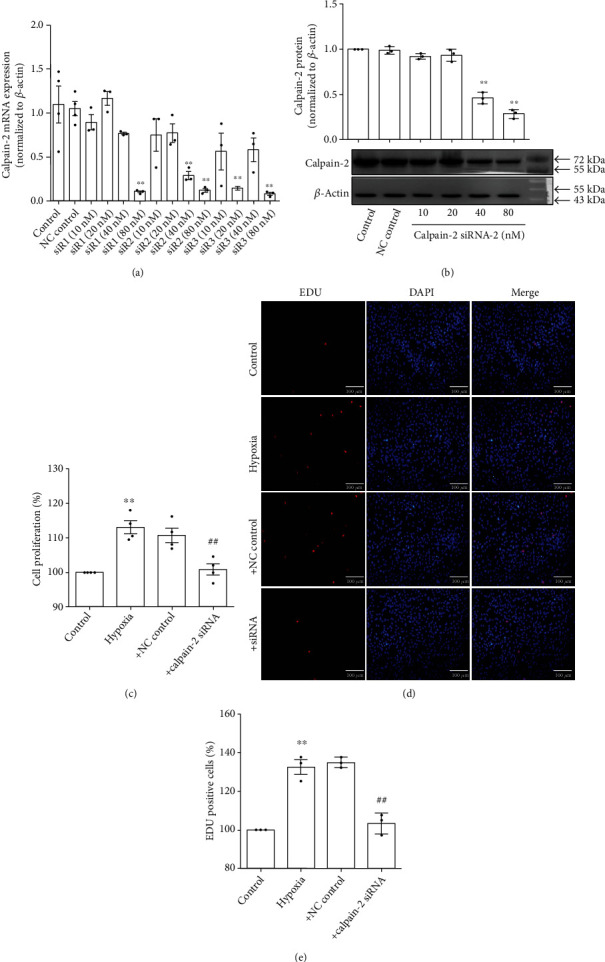
Knockdown of calpain-2 inhibited hypoxia-induced proliferation of PASMCs. (a) PASMCs were transfected with calpain-2 siRNA, and the mRNA expression of calpain-2 was detected by real-time PCR (*n* = 3). (b) PASMCs were transfected with calpain-2 siRNA, and the protein expression of calpain-2 was detected by Western blot (*n* = 3). (c) PASMCs were transfected with calpain-2 siRNA (40 nM), and the proliferation of PASMCs was detected by MTS assay (*n* = 4). (d) PASMCs were transfected with calpain-2 siRNA (40 nM), and the proliferation of PASMCs was detected by EDU staining. (e) Statistic diagram of EDU staining (*n* = 3). The data are presented as means ± S.E.M.; ^∗∗^*p* < 0.01 vs. control and ^##^*p* < 0.01 vs. hypoxia.

Inhibition of calpain-2 has been shown to attenuate proliferation of PASMCs induced by PH mediators (platelet-derived growth factor [PDGF], serotonin [5-HT], and interleukin 6 [IL-6]) [17, 18]. In this study, we therefore used the calpain-2 small interfering RNA (siRNA) to knock down the expression of calpain-2 mRNA to explore whether calpain-2 mediates hypoxia-induced PASMC proliferation. Different fragments and different concentrations of calpain-2 siRNA were transfected into PASMCs, resulting in the decrease of calpain-2 mRNA and protein expression in PASMCs, especially the effect of fragment 2 of calpain-2 siRNAs in a concentration-dependent manner (Figures 6(a) and 6(b)). Then, we used the fragment 2 of calpain-2 siRNAs at the concentration of 40 nM for the subsequent experiments. The MTS and EDU assay showed that knockdown of calpain-2 inhibited hypoxia-induced proliferation of PASMCs (Figures 6(c)–6(e)).

## 4. Discussion

This study represents the first evidence of the role of miR-137 in mediating hypoxia-induced proliferation of PASMCs, thereby potentially contributing to pulmonary arterial remodeling in PH. The main findings of the present study are as follows: (1) miR-137 was downregulated in pulmonary arteries of hypoxic PH rats and hypoxia-treated PASMCs; (2) miR-137 mimic inhibited hypoxia-induced proliferation of PASMCs by targeting calpain-2, and miR-137 inhibitor induced the proliferation of PASMCs under normoxia; (3) knockdown of calpain-2 by siRNA suppressed hypoxia-induced proliferation of PASMCs.

Hypoxia is one of the commonest causes of PH [19]. Hypoxia not only causes vasoconstriction by activating voltage-gated calcium channels resulting to increased cytosolic calcium of PASMCs, but also leads to pulmonary vascular remodeling by activating rho kinase and hypoxia-inducible factor- (HIF-) 1*α* [20]. Hypoxia also compels the differential expression of miRNAs through response elements in their promoters of HIF-1 or through indirect hypoxia-associated stimulus [21]. The role of several miRNAs including miR-206 [22], miR-130/301 [23], miR-103/107 [24], miR-150 [25], miR-let-7g [6, 10], miR-17/92 [26], miR-92b-3p [27], miR-204 [28], and miR-27a [29] in hypoxic pulmonary arterial remodeling has been reported. The present study found for the first time that miR-137 was downregulated in pulmonary arteries of hypoxic PH rats and hypoxia-treated PASMCs. Studies have reported that the downregulation of miR-137 expression is caused by the ubiquitous in hypoxic-microenvironment [30], and that miR-137 is silenced by methylation and reduction of hypermethylation of the miR-137 promoter by inhibiting DNA methyltransferase which promotes its reexpression in hypoxia condition [31, 32]. In our setting, whether these potential mechanisms are involved in hypoxia-induced, the downregulation of miR-137 expression needs further investigation.

In a variety of cancer cells, miR-137 is significantly downregulated, and transfection of miR-137 mimic to restore miR-137 expression results in significant inhibition of cell proliferation, migration, and epithelial-mesenchymal transition [11, 12, 33]. miR-137 also regulates nervous system development and synaptic plasticity [13, 34]. In high glucose-induced human umbilical vein endothelial cell injury, miR-137 is significantly upregulated and inhibition of miR-137 inhibits oxidative stress and cell apoptosis [35]. In PDGF-induced proliferation of vascular smooth muscle cells, miR-137 is significantly downregulated and overexpression of miR-137 suppresses the cell proliferation and migration by suppressing the activity of mTOR/Stat3 signaling [36]. As we described above, excessive proliferation of PASMCs is the most important cause of pulmonary vascular remodeling in PH [37]. In this study, we for the first time found that miR-137 mediated the pathogenesis of hypoxic PH by inhibiting the proliferation of PASMCs. However, the destruction of vascular intima after vascular endothelial cell injury is usually the starting point of cardiovascular diseases. In the process of PH, apoptosis, necrosis, and endothelial to mesenchymal transition occur in pulmonary arterial endothelial cells [38]. Therefore, the role of miR-137 in pulmonary arterial endothelial functions also deserves to be further studied.

miRNAs bind to the 3′-UTR of target genes, resulting in inhibition of the target genes, to participate in physiological process and the pathogenesis of diseases. Bioinformatic analysis suggests that a potential binding element for miR-137 is contained in the 3′-UTR of calpain-2. Studies have demonstrated that miR-137 binds to 3′-UTR of calpain-2 to inhibit the expression of calpain-2 [14, 16, 39]. In this study, miR-137 also suppressed the translation of calpain-2 mRNA by binding to its 3′-UTR, suggesting that the calpain-2 is a direct target of miR-137 in hypoxia which induced the proliferation of PASMCs. Moreover, as we described above, miR-137 mediates the PDGF which induced the proliferation of VSMCs by regulating the activity of mTOR/Stat3 signaling. Stat3 has been demonstrated as a key mediator of PH pathology, and the inappropriate Stat3 activation in PH has been linked to miRNA expression, such as miR-204 and miR-17/92 [40]. Therefore, whether not only calpain-2 but also Stat3 participates in the proliferation of PASMCs mediated by miR-137 in hypoxic PH or other category of PH needs further investigation.

**Figure 7:**
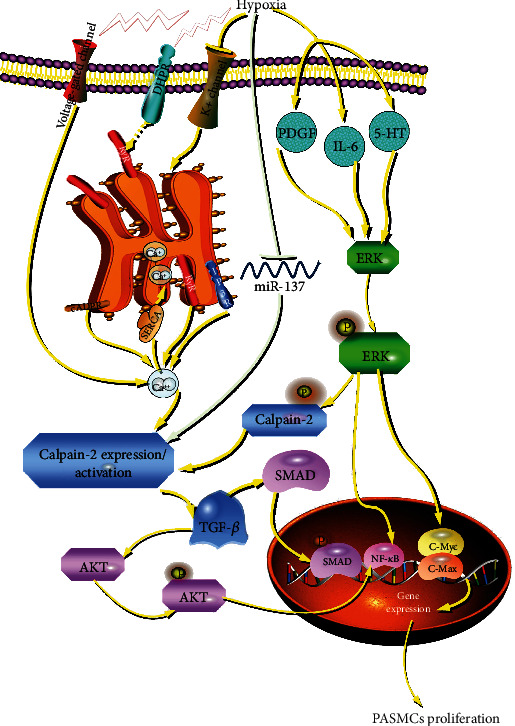
Schematic diagram of the role of miR-137 in the proliferation of PASMCs during hypoxia-induced pulmonary hypertension. Our study for the first time demonstrated that miR-137 is a novel regulator of proliferation of PASMCs in hypoxia-induced pulmonary hypertension by targeting calpain-2 pathway. DHPR: dihydropyridine receptor; RyR: ryanodine receptor; cADPR: CD38-cyclic ADP-ribose; IP3R: inositol 1,4,5-trisphosphate receptor; SERCA: sarco (endo) plasmic reticulum calcium ATPase.

Calpain-2 (m-calpain) belongs to calpain family, which is activated by hypoxia-induced intracellular calcium fluxes. Our previous study found that calpain-1/2/4 expression was increased in pulmonary arteries of hypoxic PH rats, and the specific calpain inhibitor MDL28170 inhibited hypoxia-induced PASMC proliferation [7]. Others have also reported that global knockout or smooth muscle specific knockout of calpain-4 and MDL28170 prevent pulmonary vascular remodeling of MCT- or hypoxia-induced PH and EGF- and PDGF-BB-induced cell proliferation of PASMCs [8, 17, 18]. In this study, knockdown of calpain-2 by siRNA inhibited hypoxia-induced proliferation of PASMCs. Bioinformatic analysis showed that calpain-1/4 may be not targets of miR-137 (data not shown). Notably, calpain-1 has been implicated strongly in cell motility and adhesion, while calpain-2 has been implicated strongly in cell proliferation [41]. Emerging evidence has suggested an important role of calpain-2 in proliferation of PASMCs. In hyperproliferated PASMCs treated with PH mediators (PDGF, 5-HT, and IL-6), the extracellular signal-regulated kinase (ERK) 1/2 activated calpain-2 through phosphorylation of calpain-2 at Ser50 and ERK-1/2 inhibitor PD98059 or knockdown of calpain-2 prevented calpain activation, resulting in inhibition of proliferation of PASMCs [21, 42]. In this study, we demonstrated that miR-137 mimic reduced the expressions of calpain-2, but not measured the activity of calpain-2. However, there is a study showing that miR-137 mimic pretreatment effectively prevented the oxygen-glucose deprivation and reperfusion-induced [Ca^2+^] increase, whereas the miR-137 inhibitor aggravated the [Ca^2+^] increase [39]. Given that increased intracellular [Ca^2+^] levels can activate calpain-2, we speculate that miR-137 mediates the activation of calpain-2 by regulating the concentration of [Ca^2+^] in hypoxic PH. Calpain-2 upregulated Akt phosphorylation via an intracrine transforming growth factor-*β* 1 (TGF-*β*1)/mammalian target of rapamycin complex 2 (mTORC2) mechanism, resulting in proliferation of PASMCs treated with PDGF [17]. Intracrine TGF-*β*1 pathway is initiated by calpain-mediated cleavage and activation of latent TGF-*β*1 in the Golgi complex [8]. Study has reported that bone morphogenetic protein 4 (BMP4) inhibits PDGF-stimulated calpain activation and subsequent intracrine TGF-*β*1-Smad 2/3 pathway in PASMCs [43]. All findings suggest that calpain-2 is expected to be a potential therapeutic target for proliferation of PASMCs, further for PH (Figure 7).

In the present study, we found that calpain-2, as a target of miR-137, was upregulated with the downregulation of miR-137 in hypoxic PH. However, studies have also demonstrated that protein level of calpain-2 is regulated by miR-223 acting directly on the 3′-UTR of calpain-2 mRNA as well as by miR-145, which acts via an increase in histone deacetylase 2, and histone deacetylase 2 transcriptionally inhibits calpain-2 expression by hyperacetylation of the promoter of calpain-2 gene in endothelial cells [9, 44]. Therefore, whether there also exist other miRNAs targeted calpain-2 to participate in the proliferation of PASMCs in hypoxic PH or other category of PH needs further investigation. Furthermore, besides calcium channels, potassium channels have also been reported to regulate calpain activity. Potassium channel dysfunction in PASMCs is a hallmark of PH. Transient transfection of a Kv channel or a K^+^ channel activator increases K^+^ efflux to enhance PASMC death [45, 46]. The decrease of K^+^ channel expression, such as Kv1.5 and Kv1.2, leads to the proliferation of PASMCs [47]. The opening of potassium channels promotes cell membrane hyperpolarization and reduces calcium overload. In a hypoxic environment, cell membrane depolarization inhibits the opening of potassium channels, which in turn promotes the increase of cytoplasmic free calcium concentration resulting in calpain-2 activation [48]. Therefore, the activation of calpain-2 may also be involved in the potassium channel-mediated proliferation of PASMCs.

In spite of the foregoing important findings, the present study has indeed some limitations. Transgenic or gene knockout animals of miRNA-137 and calpain-2 need to be introduced to further prove the in vivo functions of miRNA-137 and calpain-2 in pulmonary vascular remodeling and further confirm the inhibitory effect of miR-137 in hypoxia-induced proliferation of PASMCs by targeting calpain-2.

In conclusion, the present study for the first time demonstrated that hypoxia-induced downregulation of miR-137 promoted PASMC proliferation by targeting calpain-2. miR-137, a new miRNA involved in proliferation of PASMCs, further in pulmonary vascular remodeling of PH, would be a novel potential therapeutic target for PH.

## Data Availability

The research data used to support the findings of this study are available from the corresponding author upon request.
